# Comparative Genomic Analysis of ST131 Subclade C2 of ESBL-Producing *E. coli* Isolates from Patients with Recurrent and Sporadic Urinary Tract Infections

**DOI:** 10.3390/microorganisms11071622

**Published:** 2023-06-21

**Authors:** Daniel Jaén-Luchoro, Arezou Kahnamouei, Shora Yazdanshenas, Anna Lindblom, Emma Samuelsson, Christina Åhrén, Nahid Karami

**Affiliations:** 1 Institute of Biomedicine, Department of Infectious Diseases, University of Gothenburg, 40530 Gothenburg, Sweden; anna.u.lindblom@vgregion.se (A.L.); christina.ahren@vgregion.se (C.Å.); nahid.karami@microbio.gu.se (N.K.); 2 Centre for Antibiotic Resistance Research (CARe), University of Gothenburg, 40530 Gothenburg, Sweden; 3 Department of Life Sciences and Systems Biology, University of Turin, 10124 Turin, Italy; arezou.kahnamouei@edu.unito.it; 4 Sahlgrenska University Hospital, Department of Clinical Microbiology, Region Västra Götaland, 41345 Gothenburg, Sweden; 5 Sahlgrenska University Hospital, Department of Clinical Genetics and Genomics, Region Västra Götaland, 41345 Gothenburg, Sweden; emma.k.samuelsson@vgregion.se; 6 Swedish Strategic Program against Antimicrobial Resistance (Strama), Region Västra Götaland, 40544 Gothenburg, Sweden

**Keywords:** recurrent urinary tract infection, UTI, extended-spectrum beta-lactamase, ESBL, ST131 subclade C2, whole genome sequencing, *E. coli*

## Abstract

The global emergence of extended-spectrum beta-lactamase-producing *Escherichia coli* (ESBL-*E. coli*), mainly causing urinary tract infections (UTI), is a major threat to human health. ESBL-*E. coli* sequence type (ST) 131 is the dominating clone worldwide, especially its subclade C2. Patients developing recurrent UTI (RUTI) due to ST131 subclade C2 appear to have an increased risk of recurrent infections. We have thus compared the whole genome of ST131 subclade C2 isolates from 14 patients with RUTI to those from 14 patients with sporadic UTI (SUTI). We aimed to elucidate if isolates causing RUTI can be associated with specific genomic features. Paired isolates from patients with RUTI were identical, presenting 2-18 single nucleotide polymorphism (SNP) differences for all six patients investigated. Comparative genomic analyses, including virulence factors, antibiotic resistance, pangenome and SNP analyses did not find any pattern associated with isolates causing RUTI. Despite extensive whole genome analyses, an increased risk of recurrences seen in patients with UTI due to ST131 subclade C2 isolates could not be explained by bacterial genetic differences in the two groups of isolates. Hence, additional factors that could aid in identifying bacterial properties contributing to the increased risk of RUTI due to ESBL-*E. coli* ST131 subclade C2 remains to be explored.

## 1. Introduction

Extra-intestinal pathogenic *Escherichia coli*, or ExPEC, is a major human pathogen and is the most common cause of urinary tract infections (UTIs) and bloodstream infections [1,2]. Recurrent UTIs (RUTIs) affect approximately one-third of patients with UTI and thus pose an important clinical problem [3,4].

A specific ExPEC clone, sequence type 131 (ST131), is a globally spread multidrug-resistant clone with resistance to fluoroquinolones and producing CTX-M beta-lactamase, (mainly *bla*_CTX-M-15,_ *bla*_CTX-M-14_ and *bla*_CTX-M-27_) [5]. It is one of the major extended-spectrum beta-lactamase-producing *Escherichia coli* (ESBL-*E. coli*) lineages among human pathogens worldwide. ST131 is divided into three major clades: A, B and C [6]. Clades A and B are minor subsets and are associated with *fimH*41 and *fimH*22, respectively. Clade C, associated with *fimH*30, represents two subclades, C1 (also named *fimH*30R) and C2 (also named *fimH*30Rx). ST131 subclade C2 (hereafter named ST131-C2) mostly harbours *bla*_CTX-M-15_ and represents the dominant population among clade C [7].

Compared to the highly conserved core genome, there is a wide plasticity in the accessory genome of ST131, resulting in the appearance of different virotypes and plasmid contents [8]. Subpopulations of ST131-C2 have been discussed in literature. For example, a subpopulation of ST131-C2 with increased virulence, carriage of *pap*GII, increased antimicrobial resistance and chromosomally integrated *bla*_CTX-M-15_ has been described [9,10]. These characteristics of ST131-C2 isolates may lead to a severe and difficult-to-treat infections [9]. Another specific virotype of ST131-C2, with higher rates of aminoglycoside resistance and virulence gene content, shows the shifting dynamics of this pandemic clone in response to antibiotic selection pressure by establishing subsets with high survival potential [11].

We have reported that in the great majority (97%) of RUTIs due to ESBL-*E. coli*, the same strain caused almost all subsequent UTI recurrences for at least six months [12]. We also found that *fimH*30Rx, that is, ST131-C2, isolates are associated with an increased number of UTI recurrences. In a subsequent prospective study of RUTI due to ESBL-*E. coli*, we recently reported that the ST131-C2 clone dominated among ST131 isolates and was more common in patients with RUTI than in those with sporadic UTI (SUTI) (28% vs. 13%) [13]. Additionally, multivariate analysis showed a twofold increased risk for recurrences in patients infected with these isolates as compared to non-ST131 isolates.

To further evaluate if the risk of recurrences can be associated with a certain subpopulation of ST131-C2 or isolates with specific genomic traits or properties among those causing RUTI, we aimed to compare the whole genome of the ST131-C2 isolate from patients with recurrent and sporadic UTI identified in our previous study [13]. Identifying a particular genetic trait may aid in the prediction of RUTI and improve diagnostics and care of patients with a risk of developing recurrences when infected with this emerging pathogen.

## 2. Materials and Methods

### 2.1. Patients and Isolates

The 28 patients included in this study were part of a previous study investigating the bacterial features of 297 isolates from patients with recurrent as compared to sporadic UTI due to ESBL-*E. coli* in the Västra Götaland region in western Sweden [13]. In the previous study, between 1 October 2017 and 1 October 2018 all urinary ESBL-*E. coli* isolates from patients ≥15 years were collected from all clinical microbiology laboratories in the region. The laboratory databases were searched for urine samples positive for ESBL-*E. coli* in in- and outpatient settings. Epidemiological and clinical data linked to the index ESBL-*E. coli* isolates were extracted from referral data as well for all subsequent UTI episodes with ESBL-*E. coli* for one year following the index episode. Only patients with no previous recorded history of ESBL-producing bacteria in any type of clinical or screen culture were included in the study. Only voided samples with a significant number of ESBL-*E. coli* (≥10^5^ CFU/mL) in monoculture were included. Cultures with isolates of more than one bacterial species were excluded, as were cultures with referral data indicating control purposes or presence of a urinary catheter or urinary abnormalities. Patients who died or moved from the region during the year following their first infection were excluded.

Recurrent UTI was defined according to the clinical international definition (https://uroweb.org/guidelines/urological-infections (accessed on 1 June 2023)), that is, ≥2 infections within six months or at least three within one year with at least 30 days between each episode. If a urine culture with other uropathogens than ESBL-*E. coli*, including *E. coli* not producing ESBL, was detected between ESBL-*E. coli* RUTI episodes, the patient was not included. Sporadic UTI was defined as only one UTI ESBL-*E. coli* episode during one year of follow up.

*E. coli* isolates were identified according to routine clinical microbiology practice and phenotypic antibiotic resistance was determined using the disc diffusion method and breakpoints according to European Committee on Antimicrobial Susceptibility Testing (EUCAST) (https://www.eucast.org (accessed on 1 June 2023)) at the time. Cephalosporin-resistant isolates were screened for the ESBL phenotype using the double-disc diffusion assay and later genetically confirmed. For further methodological details, we refer the reader to our previous manuscript [13].

Out of the 50 ST131-C2 isolates detected in the previous study, almost all those causing RUTI (14/19) and half of those causing SUTI (14/31) were included in this study, aiming to match included patients with RUTI with those with SUTI by age and sex as increasing age was found to also increase the risk of RUTI in a previous study [13]. In addition, both the index and the subsequent isolate in six arbitrarily selected patients with RUTI were compared.

### 2.2. Whole Genome Sequencing and Assembly

DNA was extracted from fresh biomass, following a previously described protocol [14]. DNA samples were quantified with the Qubit^®^ 2.0 fluorimeter and the Qubit^TM^ dsDNA BR kit (Thermofisher Scientific, Waltham, MA, USA). Quality was determined by analysis of ratios 260/230 and 260/280 on a NanoDrop ND-1000 spectrophotometer (Thermofisher Scientific, Waltham, MA, USA). Estimation of the distribution of DNA fragment sizes was performed, using a TapeStation 2200 (Agilent Technologies, Santa Clara, CA, USA). Whole genome sequencing was performed externally, using an Illumina NovaSeq 6000 S4 (read mode 2x 150 bp) (Eurofins Genomics, Ebersberg, Germany). All strains were additionally sequenced in-house, using a MinION Mk101B sequencer (Oxford Nanopore Technologies, Oxford, UK), for the generation of longreads. Libraries were prepared, using the rapid barcoding sequencing kit (SQK-RBK004) (Oxford Nanopore Technologies, Oxford, UK) and the sequencing was performed using a FLOW-MIN106 vR9.4 (Oxford Nanopore Technologies, Oxford, UK). The sequencing run was performed for 72 h on MinKNOWN software v4.3.12 (Oxford Nanopore Technologies, Oxford, UK). Reads were basecalled and demultiplexed afterwards using Guppy v6.0.1 (Oxford Nanopore Technologies, Oxford, UK). Hybrid assemblies were performed using Unicycler v0.4.8 [15], combining short- and longreads generated by the Illumina and nanopore sequencing platforms, respectively. Quality of the genomes was assessed using the online Quality Assessment Tool (QUAST) [16]. Genomes were annotated using Prokka v1.14.6 [17] for downstream analysis and using the Prokaryotic Genome Annotation Pipeline (PGAP) for submission to GenBank [18].

### 2.3. Genomic Characterization of the Isolates

Genome sequences generated characterized by in silico multi-locus sequence typing (MLST), serotyping and phylogroup typing. MLST was determined using the software mlst 2.19.0 (https://github.com/tseemann/mlst). Serogroups were analysed using ectyper v1.0.0 [19], and phylogroups were inferred using EZclermont v0.6.3 [20]. Determination of *fimH* type was performed using FimTyper v1.1 [21]. Determination of antimicrobial resistance genes and virulence factors was performed using the Comprehensive Antibiotic Resistance Database (CARD) [22], and the Virulence Factors Database (VFDB) [23], respectively, with the software abricate v1.0.1 (https://github.com/tseemann/abricate).

### 2.4. Pan and Core Genome Analysis

The pangenome was calculated as previously described [24], using the protein sequence file obtained in the previous annotation with Prokka. Clusters of homologous proteins identified were used to build a matrix of the presence/absence of proteins. This matrix was used for clustering analysis, generating a heatmap of the presence/absence of proteins with the ComplexHeatmap package in Rstudio v1.2.5033 (http://www.rstudio.com) [25].

Additionally, core genome single-nucleotide polymorphism (SNP) analysis and SNP-core tree were performed using snippy v4.6.0 (https://github.com/tseemann/snippy ). Core genome MLST (cgMLST) analysis and cgMLST tree were performed using the online tool cgMLSTFinder v1.2 from the Center for Genomic Epidemiology (http://www.genomicepidemiology.org/), using the *Escherichia coli* (Enterobase) database [26,27].

Gene–trait association analysis through a microbial pan-GWAS was performed, using Scoary v1.6.16 [28]. The traits were “recurrent infection” (R) and “sporadic infection” (S). Input files for Scoary were generated by an additional pangenome calculation performed with Roary v3.13.0 [29], using the default parameters. The same analysis but based on SNPs was performed, using as input the SNP presence absence matrix previously generated with snippy.

## 3. Results and Discussion

A total of 28 ST131 subclade C2 ESBL-*E. coli* isolates from 14 patients with RUTI and 14 patients with SUTI were compared in the present study (Table 1). The subsequent recurrent isolate from six patients with RUTI were also included. All isolates were whole-genome sequenced by Illumina (short-read) and nanopore (long-read) technologies. The objective was to obtain the complete and closed genome of all isolates in order to have the entire genetic information for a complete comparison. The recurrent isolates from six patients with RUTI were sequenced with Illumina only and the data obtained were used to compare the first and second isolate of the respective patient. Accession numbers for Illumina and nanopore data of each isolate are included in Table S1.

### 3.1. Genome-Based Typing

Genome assembly generated 28 whole genome sequences, from which 23 were completely closed and 5 were high-quality draft genomes. Those five isolates (R1, R5, S1, S10 and S11) for which only a draft genome was obtained were re-sequenced to obtain additional long-read information for completing the genome. However, the additional reads obtained were not enough to fulfil the purpose. Basic quality indicators, annotation results and accession numbers are included in Tables S2 and S3.

With the genomes assembled, checked, and characterized, they were compared genomically to identify the relatedness among strains and specific traits that could be linked to the recurrency of the infection, i.e., a particular trait that can identify and distinguish between R and S isolates. Other studies have been performed to identify traits associated with infection recurrency, with no conclusive results [31,32,33].

Firstly, genome sequences were analysed through several typing tools for sequence typing, serotyping, phylogroup typing and *fimH*-typing, to confirm the previous experimental determinations of these parameters. There was a 100% correlation; all isolates belonged to the ST131-C2, phylogroup B2 and serotype O25:H4, and were of *fimH*30 type.

Secondly, we investigated their identity by SNP analyses between the index and subsequent recurrent isolate in six patients with RUTI. The differences in the coupled isolates varied from 2 to 18 SNPs. (Table S4). We could thus confirm our previous findings [12] and findings from others that the same strain reoccurs, causing the subsequent UTI episode [33,34], most likely by reintroduction from the faecal or vaginal flora or from persistent intrabladder colonisation after the first infection [13,34,35]. Accordingly, it will be sufficient to analyse the patients’ index isolates when comparing R and S isolates. It also suggests that a change in strain properties from the first to the subsequent UTI does not explain the occurrence of the recurrence event.

Thirdly, the 28 R and S isolates were compared for various genetic properties as outlined below.

### 3.2. Virulence and Antibiotic Resistance Determinants

Genomes were analysed to identify all possible encoded virulence factors (VF). The objective was to determine the possible differential distribution of VF, which could be related to the capacity to develop a recurrent infection, or the lack thereof. A total of 98 VF or virulence determinants were identified using the VFDB, from which 67 VFs were present in almost all isolates (or missing only in one of the isolates) (Table S5). The remaining 31 VFs investigated were distributed differentially among the different isolates (Table 2). Even though some virulence factors have been indicated to be over-represented in RUTIs in previous studies [36], other studies have indicated that R and S isolates are similar in terms of VF and that no virulence traits can be associated with recurrency [33,37]. For example, Ejrnæs et al. indicated that 12 VF had a higher prevalence in isolates causing relapse or persistent infections [38]. The analysis with the VFDB showed that, from the 12 VFs indicated by the mentioned study, seven were not found in any of the isolates studied in the present work (*sfa/focDE*, *iroN*, *KpsM II*, Kpsm II K2, *malX*, *usp*, *agn43* and *PapA*), *PapH* was present in 20 of them, and *ChuA* and *FyuA* were present in all. Additionally, from all the virulence factors identified in our study, some predominance of certain VFs in one of the two subsets of isolates was noted. For example, *hlyABCD* is mostly, but not exclusively, present in S isolates (9/14) rather than R isolates (5/14), while cnf1 is predominant in R isolates (9/14) rather than S isolates (4/14). None the less, we found no distribution pattern particularly linked to the R or S isolates, but rather a random distribution of the VF between both types of isolates, which has also been reported previously [38]. Nor did we find any association according to the virotypes described by Blanco et al. [30], based on presence or absence of the operon *afa*, the siderophore *iroN*, the brain endothelium invasion factor *ibeA*, and the secreted autotransporter toxin sat; 24 of the isolates were classified as virotype C (*afa* negative, iroN negative, *ibeA* negative, and *sat* positive), which has been indicated as the most predominant type [30]. For the remaining four isolates, three recurrent isolates (R7, R9, R11) were virotype A (*afa* positive, *iroN* negative, *ibeA* negative, and sat positive); and one sporadic isolate (S3) did not match any of the four virotypes A-D. Nor did we find associations to RUTI when analysing the 55 VFs and virulence scoring for extraintestinal *E. coli* described by Johnsson et al. [39]. Twelve of the VFs listed in the aforementioned study were already found by the previous BLAST analysis with the VFDB. primers provided by Johannson et al. [39] for the 55 VFs used for in silico PCR analysis, identifying five extra VFs (*papG* allele II, *yfcV*, *hra*, *kpsM II*, and *kfiC* (*K5*)) that showed no distribution correlated to S or R isolates. Altogether, these results reinforce the statement that recurrency may not be linked to a particular VF trait, but instead as suggested by others the success of ST131, the clade C may be especially associated with enhanced persistence within its host, evading host defence and antibiotic clearance [7,33,40,41,42].

Since recurrency of infection has been previously linked to antibiotic-resistance traits [43], the same approach applied for VF in the present work was undertaken focused on antibiotic-resistance genes. In this case, 64 antibiotic-resistance-related genes in the CARD were detected, from which 50 were present in all isolates or absent in almost isolates (Table S6). The remaining 14 resistance genes were differentially distributed among the isolates but mixed between R and S strains (Table 3), with no clear differentiation between the two types of isolates. Similarly, to VF, some antibiotic resistance genes seem to be more present in one type of isolate. In this sense, *mphA*, sul1 and *dfrA17* are present in 9/14 S isolates, while is only present in 4/14, 5/14 and 2/14 R isolates, respectively. In any case, as stated previously, the distribution of the genes does not point to a strong link to R or S isolates in the group of strains studied.

McNully et al. also identified an enrichment of variants in anaerobic metabolism genes in *E. coli* ST131 clade C [42]. We could not obtain publicly available accession numbers or locus tags for each gene highlighted in this study. Because of that, we were only able to correlate four genes by name, which presented at least three alleles in the mentioned study, and analyse them in the present work (*aroD*, *eutB*, *nemA*, *nirB*) under the hypothesis that different alleles could be present only in R or S isolates. The result showed 100% identity between sequences of both kind of strains. Even though this result, a more in-depth analysis should be performed with more genes involved in anaerobic metabolism to obtain a more comprehensive result in this aspect.

### 3.3. Pangenome Analysis

**Figure 1:**
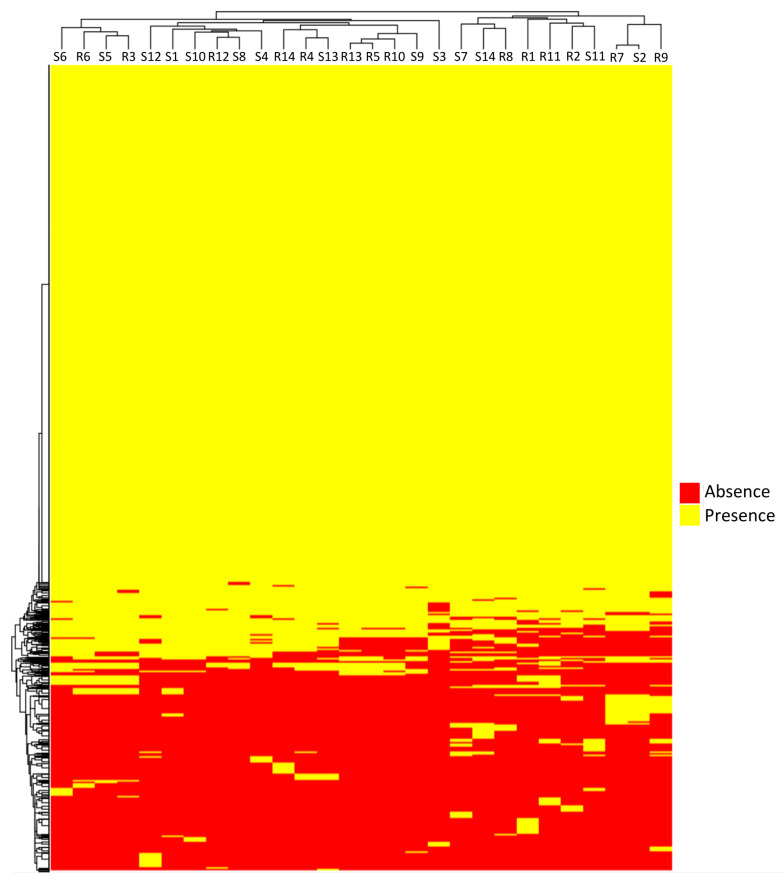
Clustering presence/absence of proteins based on the pangenome matrix in 28 ESBL-*E. coli* isolates from patients with recurrent (R) or sporadic (S) urinary tract infections.

A pangenome was calculated, using all 28 genome sequences for the index isolates included in this study. The pangenome revealed a total of 6,432 homologous protein clusters (Figure S1). The pangenome matrix, indicating the presence or absence of a particular cluster in a particular isolate, was used for clustering analysis. The hypothesis was that differential distribution of genes conforming to the pangenome could separate the two kinds of isolates, and that the functions developed for these specific proteins could give clues about the capacity of an isolate to cause recurrent UTI. The heatmap generated showed no differential distribution of R and S isolates, indicating that there was not a particular pattern of presence/absence of genes associated with R or S isolates (Figure 1).

Accessory genes were also investigated, determining exclusive genes for each strain, that is, genes present in a particular strain but absent in the rest. In the case of R isolates, the number of exclusive genes per strain ranged from 1 to 119; while for S isolates the number ranged from 0 to 112 (Table S7). The mean of exclusive genes per strain was 30.8 for R isolates, and 27.6 in S isolates. The variation in the number of exclusive genes as well as the similar mean of exclusive genes per strain between R and S isolates may indicate that there is no correlation between the capacity to accumulate differential genes and the ability to cause recurrent infections. Genes present only in all R isolates but not in S isolates, and vice versa, were also studied. No exclusive genes present neither only in R nor S isolates were found.

**Figure 2:**
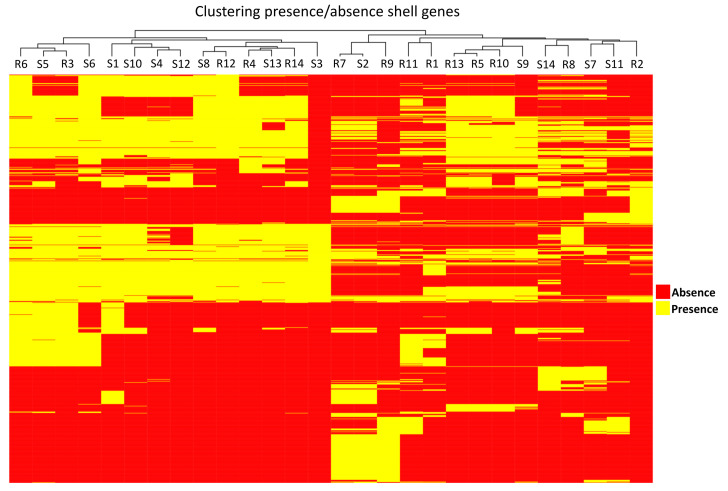
Clustering analysis based on presence/absence of genes which have been annotated in <5% and >95% of the genomes studied, i.e., intermediate genes present in 28 ESBL-*E. coli* isolates from patients with recurrent (R) or sporadic (S) urinary tract infections.

McNally et al. established a strong correlation between the distribution of intermediate frequency genes (present in >5% and <95% of the genomes studied) and sequence clusters [42]. We speculated about the possibility of a correlation of the distribution of these genes and the ability of an isolate to cause recurrent infections. A total of 920 intermediate frequency genes were identified, and clustering analysis based on these genes among the 28 isolates studied did not show a clear separation between S and R isolates (Figure 2).

A more in-depth analysis from the pangenome point of view was also performed, using a microbial pan-GWAS approach, trying to establish a gene–trait association, being in this case defined as a trait representing R or S isolates. The objective was to find a gene, or group of genes, strongly associated with a particular trait, i.e., a gene or group of genes predominantly, or completely, associated with R or S isolates. When R was considered the reference trait, the two top-ranked proteins identified were dihydrofolate reductase type 1 and streptomycin 3′-adenylyltransferase, for which a sensitivity of 14.29% and a specificity of 35.71% (odds ratio: 0.09; *p*-value: 0.02) were calculated. The same calculations using S as a reference trait were done, and indicated, for these same top-ranked proteins, a sensitivity and specificity of 64.29% and 85.71%, respectively (odds ratio: 10.80 and *p*-value: 0.02). The obtained percentages do not allow for an interpretation of the importance these two proteins could have in terms of recurrency or sporadic infections. This means that the results showed no clear gene–trait association that could determine if, when that gene was found, a particular isolate would represent a potential R or S isolate, even though percentages are higher when S is taken as a reference trait. The same analysis was performed, but focusing on SNP, i.e., if a particular SNP (or group of SNPs) could be linked to S or R isolates, with no results obtained in terms of SNPs strongly linked to a particular trait.

### 3.4. SNP Analysis

**Figure 3:**
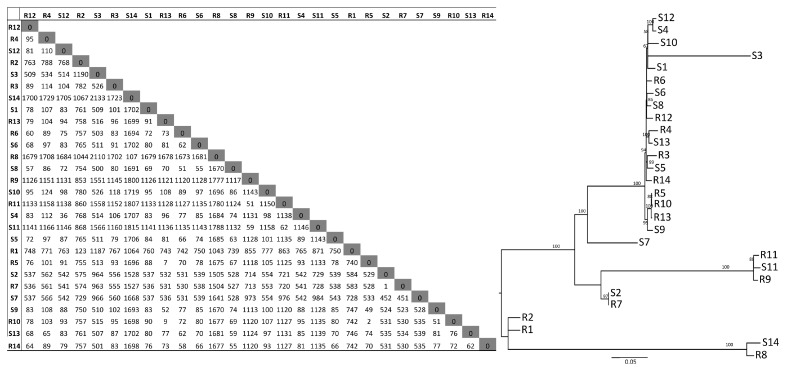
SNP core analysis performed on 28 ESBL-*E. coli* isolates from patients with recurrent (R) or sporadic (S) urinary tract infections. Number of SNPs between the different isolates, as well as the SNP-core tree, are shown.

A SNP-core tree was also determined, assessing the identify differences between R and S isolates based on SNP-phylogeny (Figure 3). Differences ranging from 1 to more than 2000 SNPs were found among the isolates. This difference is larger than those previously reported for ST131-C2 UTI isolates, where the diversity was strikingly low [43,44]. However, the SNP analyses revealed that some isolates seem more closely related to each other than others, but this could not be explained by an epidemiological relationship between cases as a particular ward or hospital, long-term facility or a certain part of a region, contrary to what have been described by others [44,45]. The distribution of R and S isolates was mixed, showing no differences traceable to a particular kind of isolate. The difference for R isolates (1-1,780 SNPs) and for S isolates (36 -2,133) was of a similar magnitude, further indicating the lack of a particular trait in R isolates. Taken together, from the core-genome perspective, the R and S isolates included in this study cannot be differentiated by variations in the core genome. This agrees with results previously described for RUTI due to a polyclonal set of sensitive *E. coli* without ESBL, lacking however ST131 isolates [37].

**Figure 4:**
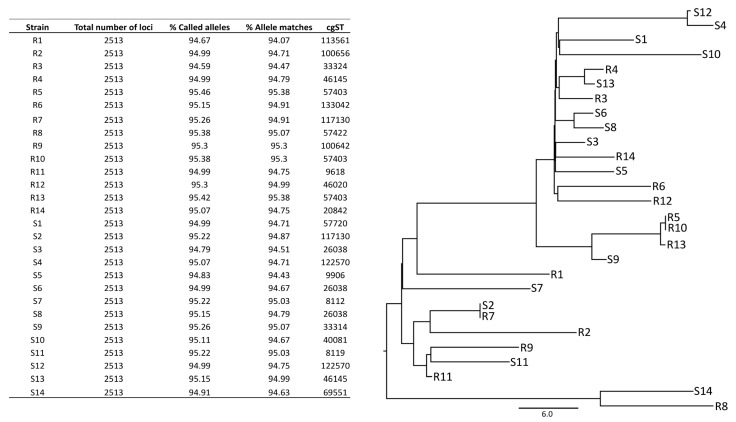
cgMLST results after analysis of the 28 isolates from patients with recurrent (R) and sporadic (S) infections. The percentages of alleles called and matched, and cgMLST tree, are indicated.

Finally, a cgMLST approach was also assessed. Of a total of 2513 loci present in the database, more than 94% were called and matched in all the isolates (Figure 4). The tree based on the cgMLST was built, showing, once more, no differential distribution of R isolates and S isolates, indicating that they cannot been distinguished by cgMLST determinations 

### 3.5. Limitations

These isolates were part of a large cohort of well-characterized isolates from patients with RUTI and SUTI due to ESBL-*E. coli* from an entire region, minimising the risk of clonal selection and epidemiologically linked isolates being included. Nonetheless, the low number of isolates in this study is a limitation. Despite this limitation, results correlate with previous studies performed with larger populations of more diverse strains, in which no genetic features were found to conclusively identify recurrency [37]. Additionally, there were no trends in dissimilarities detected in the various types of comparisons, suggesting that adding additional isolates would not alter the conclusions.

Secondly, we cannot exclude that despite presenting the same genes, the expression of a particular gene or combination of genes may vary between R and S isolates, and that this may have an impact on virulence or recurrency. From one of our previous studies, it can be seen that the same genes in different context, such as strains with different genetic backgrounds, can have different levels of expression [46]. Similarly, differences in phenotypic traits expressed in various environments and in importance in the ESBL-*E coli* UTI pathogeneses may not have been revealed by the genetic analyses. Differences in early biofilm formation and type 1 fimbriae production between clade B and C isolates of ST131 have been described, but no differences were noted between subclade C1 and C2 ST131 ESBL- *E. coli* isolates [40]. In any case, finding a sequence or genetic trait that can be easily detected and linked to recurrent isolates of the emerging ST131 C2 subclade would be of great aid in foreseeing the risk of RUTI in patients infected with these isolates. It is also important to distinguish them from isolates that do not have that capacity, and hence, can be less problematic for the patient in the long term. In this sense, the present study tries to find genomic markers linked to recurrent or sporadic ST131-C2 *E. coli* isolates using different genomic approaches (pangenome, cgMLST, SNPs, pan-GWAS, VF, antibiotic resistance). Despite extensive genomic analyses, the results have shown no particular genomic traits underlying that distinction.

## 4. Conclusions

The genomic analysis and comparisons from different points of view presented here did not find any genetic traits or SNP particularly associated with recurrency, nor any gene-based clustering that correlates with recurrent isolates and differentiates them from isolates causing sporadic urinary tract infections. The increased risk of recurrences seen in patients with UTI due to ST131-C2 isolates can thus not solely be explained by bacterial genetic differences in the two groups of isolates. Other explanations need to be searched, including the expression of genes, phenotypic traits and patient and bacterial interactions that could favour the development of recurrent UTI.

## Figures and Tables

**Table 1:** Characteristics for the 28 patients with recurrent or sporadic UTI and their studied ESBL-*E. coli* isolates.

Patient and Isolates ID	Sex	Age (Year)	HC/PC ^b^	Date of Isolation	Phenotypic Resistance to			Days to First Recurrence	Virotype ^d^
					CIP ^c^	TOB ^c^	TMP ^c^		
Recurrent UTI									
R1	M	77	PC	Oct. 2017	R	S	R	40	C
R2	M	80	HC	Oct. 2017	R	R	R	68	C
R3^a^	M	51	HC	Oct. 2017	R	R	S	42	C
R4	M	59	PC	Nov. 2017	R	R	S	158	C
R5	F	88	HC	Nov. 2017	R	R	R	58	C
R6 ^a^	F	21	HC	Dec. 2017	R	R	S	78	C
R7	M	86	HC	Feb. 2018	R	R	S	106	A
R8	F	65	HC	Mar. 2018	R	R	S	41	C
R9 ^a^	F	59	HC	May 2018	R	S	S	33	A
R10	M	78	HC	Jun. 2018	R	R	S	31	C
R11 ^a^	F	89	HC	Jun. 2018	R	R	S	62	A
R12	F	34	HC	Aug. 2018	R	R	R	73	C
R13 ^a^	F	80	HC	Sep. 2018	R	R	R	146	C
R14 ^a^	F	74	PC	Sep. 2018	R	R	R	54	C
**Sporadic UTI**									
S1	M	73	HC	Nov. 2017	R	R	R		C
S2	F	78	PC	Dec. 2017	R	R	S		C
S3	M	74	PC	Jan. 2018	R	R	S		None
S4	M	74	HC	Jan. 2018	R	R	R		C
S5	M	51	HC	Jan. 2018	R	R	R		C
S6	F	68	PC	Feb. 2018	R	R	S		C
S7	F	85	PC	Mar. 2018	R	R	R		C
S8	F	25	HC	May 2018	R	R	S		C
S9	F	69	PC	May 2018	R	R	R		C
S10	F	47	HC	May 2018	R	R	R		C
S11	F	18	HC	Jul. 2018	R	R	R		C
S12	M	84	HC	Aug. 2018	R	R	R		C
S13	F	86	HC	Sep. 2018	R	R	S		C
S14	M	63	PC	Sep. 2018	R	R	R		C

a—The second isolate from these patients was also analysed by SNP analyses. b—Index isolate detected during hospital care (HC) or in primary care (PC). c—ciprofloxacin (CIP), tobramycin (TOB), trimetoprim (TMP): R = resistant, S = sensitive. d—virotype determination according to [30].

**Table 2:** Virulence factors (VF) identified using the Virulence Factor DataBase (VFDB). Only VFs distributed differentially among the 28 ESBL-*E. coli* isolates are shown out of 98 VFs explored.

	Adhesins/Fimbria					Toxins		T2SS	Hemolysins		Others	
Isolate	afaA,B-I,C-I,D	afaE-I	papCDFGHJK	papE	fimB	senB	cnf1	gspCDEFGHIJK	hlyABD	hlyC	draP	astA
R1	.	.	Y	.	.	.	.	Y	.	.	.	.
R2	.	.	.	.	.	.	.	Y	.	.	.	.
R3	.	.	Y	.	.	Y	Y	.	Y	Y	.	.
R4	.	.	Y	.	.	.	Y	.	Y	Y	.	.
R5	.	.	Y	.	.	Y	.	.	.	.	.	.
R6	.	.	Y	.	.	Y	Y	.	Y	Y	.	.
R7	Y	Y	.	.	.	Y	.	.	.	.	Y	.
R8	.	.	.	.	Y	Y1	.	Y	.	.	.	.
R9	Y	.	.	.	.	.	.	Y	.	.	Y	.
R10	.	.	Y	.	.	.	.	.	.	.	.	.
R11	Y	.	.	.	.	.	.	Y	.	.	Y	.
R12	.	.	Y	.	.	.	Y	.	Y	Y	.	.
R13	.	.	Y	.	.	Y	.	.	.	.	.	.
R14	.	.	Y	.	.	.	Y	.	Y	Y	.	.
S1	.	.	Y	.	.	Y	Y	.	Y	Y	.	.
S2	Y	Y	.	.	.	Y	.	.	.	.	Y	.
S3	.	.	Y	.	.	.	Y	.	Y	Y	.	.
S4	.	.	Y	.	.	.	Y	.	Y	Y	.	.
S5	.	.	Y	.	.	Y	Y	.	Y	Y	.	.
S6	.	.	Y	.	.	.	Y	.	Y	Y	.	.
S7	Y	.	.	.	.	.	.	.	.	.	Y	.
S8	.	.	Y	.	.	Y	Y	.	Y	Y	.	.
S9	.	.	Y	.	.	Y	.	.	.	.	.	.
S10	.	.	Y	.	.	Y	Y	.	Y	Y	.	Y
S11	Y	.	.	.	.	.	.	Y	.	.	Y	.
S12	.	.	Y	.	.	.	Y	.	Y	Y	.	.
S13	.	.	Y	.	.	.	Y	.	Y	.	.	.
S14	.	.	Y	Y	Y	Y	.	Y	Y	Y	.	.

Y = yes, presence of the gene.

**Table 3:** Identified genes related to antibiotic resistance using CARD database. Only genes distributed differentially among the 28 isolates are shown out of 64 resistance genes explored.

	Aminoglycosides						Macrolides	β-lactams		Diaminopyrimidines		Sulfonamides		Tetracyclins
Isolate	AAC(3)-IIe	AAC(6′)-Ib-cr	APH(3″)-Ib	APH(6)-Id	aadA2	aadA5	mphA	OXA-1	TEM-1	dfrA12	dfrA17	sul1	sul2	tet(A)
R1	.	.	.	.	.	.	.	.	.	.	.	.	.	.
R2	.	Y	.	.	Y	.	Y	Y	.	Y	.	Y	.	Y
R3	Y	Y	.	.	.	.	.	Y	.	.	.	.	.	.
R4	Y	Y	.	.	.	.	.	Y	.	.	.	.	.	.
R5	.	Y	.	.	.	Y	Y	Y	.	.	Y	Y	.	Y
R6	.	Y	.	.	.	.	.	Y	.	.	.	.	.	Y
R7	.	Y	Y	Y	.	.	.	Y	.	.	.	.	Y	Y
R8	Y	Y	.	.	.	.	.	Y	.	.	.	Y	.	.
R9	.	.	.	.	.	.	.	.	.	.	.	.	.	.
R10	.	Y	.	.	.	.	.	Y	.	.	.	.	.	Y
R11	.	Y	.	.	.	.	.	Y	.	.	.	.	.	Y
R12	Y	Y	.	.	.	.	.	Y	.	.	.	.	.	.
R13	.	Y	.	.	.	Y	Y	Y	.	.	Y	Y	.	Y
R14	Y	Y	.	.	Y	.	Y	Y	.	Y	.	Y	.	Y
S1	Y	Y	.	.	.	Y	Y	Y	.	.	Y	Y	.	Y
S2	.	Y	Y	Y	.	.	.	Y	.	.	.	.	Y	Y
S3	Y	Y	.	.	.	.	.	Y	.	.	.	.	.	.
S4	Y	Y	.	.	.	.	Y	Y	.	.	Y	Y	.	Y
S5	Y	Y	.	.	.	Y	Y	Y	.	.	Y	Y	.	Y
S6	.	Y	.	.	.	.	.	Y	.	.	.	.	.	.
S7	Y	Y	Y	Y	.	Y	Y	Y	Y	.	Y	Y	Y	Y
S8	Y	Y	.	.	.	.	.	Y	.	.	.	.	.	Y
S9	.	Y	.	.	.	Y	Y	Y	.	.	Y	Y	.	Y
S10	Y	Y	.	.	.	Y	Y	Y	.	.	Y	Y	.	.
S11	.	Y	.	.	.	Y	Y	Y	.	.	Y	Y	.	.
S12	Y	Y	.	.	.	Y	Y	Y	.	.	Y	Y	.	Y
S13	Y	Y	.	.	.	.	.	Y	.	.	.	.	.	.
S14	.	.	Y	Y	.	Y	Y	.	Y	.	Y	Y	Y	Y

Y = yes, presence of the gene.

## Data Availability

The sequencing data, including raw reads and genome sequences, are available in GenBank and the Sequence Read Archive (SRA) at NCBI. Accession numbers are included in Tables S1–S3.

## Supplementary Materials

The following supporting information can be downloaded at: https://www.mdpi.com/article/10.3390/microorganisms11071622/s1, Table S1. Sequence Read Archive (SRA) accession numbers for Illumina and Nanopore Read datasets generated for each isolate; Table S2. Basic characteristics of the genome sequences obtained for all recurrent isolates; Table S3. Basic characteristics of the genome sequences obtained for all sporadic isolates; Table S4. Number of SNPs identified between the pairs of isolates from the same patient with recurrent infection (Index and subsequent isolate).
