# Supplementary material for: Comparative Genomic Analysis of ST131 Subclade C2 of ESBL-Producing E. coli Isolates from Patients with Recurrent and Sporadic Urinary Tract Infections
Source: Microorganisms. 2023 Jun 21;11(7):1622. doi: 10.3390/microorganisms11071622 (PMC10384980; doi:10.3390/microorganisms11071622)

Table S1. Sequence Read Archive (SRA) accession numbers for Illumina and Nanopore Read datasets generated for each isolate.

Isolate	Illumina reads accession number	Nanopore reads accesion number
R1	SRR22404178	SRR22404173
R2	SRR22405943	SRR22405913
R3	SRR22405942	SRR22405912
R3B*	SRR22485986	-
R4	SRR22405917	SRR22405911
R5	SRR22404177	SRR22404172
R6	SRR22405906	SRR22405910
R6B*	SRR22485985	-
R7	SRR22405938	SRR22405909
R8	SRR22405931	SRR22405908
R9	SRR22405930	SRR22405907
R9B*	SRR22485988	-
R10	SRR22405929	SRR22405905
R11	SRR22405928	SRR22405904
R11B	SRR22485987	-
R12	SRR22405926	SRR22405903
R12B*	SRR22485983	-
R13	SRR22405941	SRR22405902
R14	SRR22405927	SRR22405901
R14B*	SRR22485984	-
S1	SRR22404176	SRR22404171
S2	SRR22405925	SRR22405900
S3	SRR22405924	SRR22405899
S4	SRR22405923	SRR22405898
S5	SRR22405922	SRR22405940
S6	SRR22405921	SRR22405939
S7	SRR22405920	SRR22405937
S8	SRR22405919	SRR22405936
S9	SRR22405918	SRR22405935
S10	SRR22404175	SRR22404170
S11	SRR22404174	SRR22404169
S12	SRR22405916	SRR22405934
S13	SRR22405915	SRR22405933
S14	SRR22405914	SRR22405932

*Subsequence Isolate of the same patient.

Table S2. Basic characteristics of the genome sequences obtained for all recurrent isolates.

	R1	R2	R3	R4	R5	R6	R7	R8	R9	R10	R11	R12	R13-	R14
**Accesion n**	JAPKFR000000000	CP107188-CP107191	CP107184-CP107187	CP107182-CP107183	JAPKFQ000000000	CP107178-CP107181	CP107174-CP107177	CP107172-CP107173	CP107164-CP107171	CP107162-CP107163	CP107155-CP107161	CP107153-CP107154	CP107151-CP107152	CP107147-CP107150
**# contigs**	63	4	4	2	14	4	4	2	8	2	7	2	2	4
**Total length**	5,335,569	5,266,470	5,509,526	5,444,223	5,304,740	5,575,248	5,363,779	5,245,954	5,276,398	5,266,854	5,357,694	5,352,431	5,308,215	5,432,600
**GC (%)**	50.71	50.8	50.67	50.75	50.79	50.72	50.6	50.78	50.67	50.79	50.74	50.71	50.8	50.72
**N50**	667,722	5,095,314	5,283,900	5,325,420	1,528,023	5,297,994	5,118,568	5,096,686	5,100,590	5,128,182	5,120,131	5,228,698	5,120,420	5,230,650
**Total Genes**	5,281	5,171	5,495	5,431	5,256	5,561	5,283	5,168	5,170	5,205	5,288	5,305	5,252	5,396
**CDS**	5,177	5,058	5,386	5,318	5,145	5,448	5,166	5,051	5,055	5,092	5,175	5,192	5,139	5,283
**RNA genes (clusters)**	ND	7	7	7	ND	7	7	7	7	7	7	7	7	7
**tRNAs**	82	86	82	86	84	86	90	90	88	86	86	86	86	86
**Pseudogenes**	200	209	252	229	221	244	214	196	187	216	205	231	225	257

Table S3. Basic characteristics of the genome sequences obtained for all sporadic isolates.

	S1	S2	S3	S4	S5	S6	S7	S8	S9	S10	S11	S12	S13	S14
**Accesion N**	JAPKFP000000000	CP107143-CP107146	CP107142	CP107140-CP107141	CP107137-CP107139	CP107134-CP107136	CP107128-CP107133	CP107126-CP107127	CP107122-CP107125	JAPKFO000000000	JAPKFN000000000	CP107120-CP107121	CP107117-CP107119	CP107114-CP107116
**# contigs**	7	4	1	2	3	3	6	2	4	3	9	2	3	3
**Total length**	5,454,249	5,363,685	5127,186	5,363,539	5,506,813	5,564,723	5,388,022	5,382,852	5,248,437	5,415,538	5,261,769	5,431,930	5,402,261	5,439,943
**GC (%)**	50.61	50.6	50.68	50.71	50.68	50.68	50.86	50.7	50.8	50.67	50.78	50.67	50.72	50.78
**N50**	4,054,986	5,118,474	5,127,186	5,226,065	5,258,595	5,332,681	5,247,286	5,241,944	5,071,232	5,260,846	5,060,226	5,281,079	5,287,038	5,259,570
**Total Genes**	5,431	5,286	5,027	5,317	5,481	5,559	5,354	5,337	5,184	5,377	5,189	5,378	5,375	5,433
**CDS**	5,318	5,169	4,915	5,201	5,369	5,446	5,239	5,225	5,072	5,264	5,077	5,265	5,262	5,315
**RNA genes (clusters)**	ND	7	7	7	7	7	7	7	7	ND	ND	7	7	7
**tRNAs**	86	90	85	89	85	86	88	85	85	86	85	86	86	91
**Pseudogenes**	233	213	195	229	247	253	232	234	219	241	199	241	231	221

Table S4. Number of SNPs identified between the pairs of isolates from the same patient with recurrent infection (Index and subsequent isolate).

Patient	Ref. genome	Query genome	Nº of variants detected
P01	R9	R9B	5
P02	R11	R11B	18
P03	R3	R3B	3
P04	R6	R6B	10
P05	R14	R14B	2
P06	R12	R12B	6

Table S5. Virulence factors/determinants found in common in all isolates.

ABR factor	R1	R2	R3	R4	R5	R6	R7	R8	R9	R10	R11	R12	R13	R14	S1	S2	S3	S4	S5	S6	S7	S8	S9	S10	S11	S12	S13	S14
aslA	Y	Y	Y	Y	Y	Y	Y	Y	Y	Y	Y	Y	Y	Y	Y	Y	Y	Y	Y	Y	Y	Y	Y	Y	Y	Y	Y	Y
chuA	Y	Y	Y	Y	Y	Y	Y	Y	Y	Y	Y	Y	Y	Y	Y	Y	Y	Y	Y	Y	Y	Y	Y	Y	Y	Y	Y	Y
chuS	Y	Y	Y	Y	Y	Y	Y	Y	Y	Y	Y	Y	Y	Y	Y	Y	Y	Y	Y	Y	Y	Y	Y	Y	Y	Y	Y	Y
chuT	Y	Y	Y	Y	Y	Y	Y	Y	Y	Y	Y	Y	Y	Y	Y	Y	Y	Y	Y	Y	Y	Y	Y	Y	Y	Y	Y	Y
chuU	Y	Y	Y	Y	Y	Y	Y	Y	Y	Y	Y	Y	Y	Y	Y	Y	Y	Y	Y	Y	Y	Y	Y	Y	Y	Y	Y	Y
chuV	Y	Y	Y	Y	Y	Y	Y	Y	Y	Y	Y	Y	Y	Y	Y	Y	Y	Y	Y	Y	Y	Y	Y	Y	Y	Y	Y	Y
chuW	Y	Y	Y	Y	Y	Y	Y	Y	Y	Y	Y	Y	Y	Y	Y	Y	Y	Y	Y	Y	Y	Y	Y	Y	Y	Y	Y	Y
chuX	Y	Y	Y	Y	Y	Y	Y	Y	Y	Y	Y	Y	Y	Y	Y	Y	Y	Y	Y	Y	Y	Y	Y	Y	Y	Y	Y	Y
chuY	Y	Y	Y	Y	Y	Y	Y	Y	Y	Y	Y	Y	Y	Y	Y	Y	Y	Y	Y	Y	Y	Y	Y	Y	Y	Y	Y	Y
csgB	Y	Y	Y	Y	Y	Y	Y	Y	Y	Y	Y	Y	Y	Y	Y	Y	Y	Y	Y	Y	Y	Y	Y	Y	Y	Y	Y	Y
csgD	Y	Y	.	Y	Y	Y	Y	Y	Y	Y	Y	Y	Y	Y	Y	Y	Y	Y	Y	Y	Y	Y	Y	Y	Y	Y	Y	Y
csgF	Y	Y	.	Y	Y	Y	Y	Y	Y	Y	Y	Y	Y	Y	Y	Y	Y	Y	Y	Y	Y	Y	Y	Y	Y	Y	Y	Y
csgG	Y	Y	.	Y	Y	Y	Y	Y	Y	Y	Y	Y	Y	Y	Y	Y	Y	Y	Y	Y	Y	Y	Y	Y	Y	Y	Y	Y
entA	Y	Y	Y	Y	Y	Y	Y	Y	Y	Y	Y	Y	Y	Y	Y	Y	Y	Y	Y	Y	Y	Y	Y	Y	Y	Y	Y	Y
entB	Y	Y	Y	Y	Y	Y	Y	Y	Y	Y	Y	Y	Y	Y	Y	Y	Y	Y	Y	Y	Y	Y	Y	Y	Y	Y	Y	Y
entC	Y	Y	Y	Y	Y	Y	Y	Y	Y	Y	Y	Y	Y	Y	Y	Y	Y	Y	Y	Y	Y	Y	Y	Y	Y	Y	Y	Y
entD	Y	Y	Y	Y	Y	Y	Y	Y	Y	Y	Y	Y	Y	Y	Y	Y	Y	Y	Y	Y	Y	Y	Y	Y	Y	Y	Y	Y
entE	Y	Y	Y	Y	Y	Y	Y	Y	Y	Y	Y	Y	Y	Y	Y	Y	Y	Y	Y	Y	Y	Y	Y	Y	Y	Y	Y	Y
entF	Y	Y	Y	Y	Y	Y	Y	Y	Y	Y	Y	Y	Y	Y	Y	Y	Y	Y	Y	Y	Y	Y	Y	Y	Y	Y	Y	Y
entS	Y	Y	Y	Y	Y	Y	Y	Y	Y	Y	Y	Y	Y	Y	Y	Y	Y	Y	Y	Y	Y	Y	Y	Y	Y	Y	Y	Y
fdeC	Y	Y	Y	Y	Y	Y	Y	Y	Y	Y	Y	Y	Y	Y	Y	Y	Y	Y	Y	Y	Y	Y	Y	Y	Y	Y	Y	Y
fepA	Y	Y	Y	Y	Y	Y	Y	Y	Y	Y	Y	Y	Y	Y	Y	Y	Y	Y	Y	Y	Y	Y	Y	Y	Y	Y	Y	Y
fepB	Y	Y	Y	Y	Y	Y	Y	Y	Y	Y	Y	Y	Y	Y	Y	Y	Y	Y	Y	Y	Y	Y	Y	Y	Y	Y	Y	Y
fepC	Y	Y	Y	Y	Y	Y	Y	Y	Y	Y	Y	Y	Y	Y	Y	Y	Y	Y	Y	Y	Y	Y	Y	Y	Y	Y	Y	Y
fepD	Y	Y	Y	Y	Y	Y	Y	Y	Y	Y	Y	Y	Y	Y	Y	Y	Y	Y	Y	Y	Y	Y	Y	Y	Y	Y	Y	Y

Continuation Table S5. Virulence factors/determinants found in common in all isolates.

AB factor	R1	R2	R3	R4	R5	R6	R7	R8	R9	R10	R11	R12	R13	R14	S1	S2	S3	S4	S5	S6	S7	S8	S9	S10	S11	S12	S13	S14
fepG	Y	Y	Y	Y	Y	Y	Y	Y	Y	Y	Y	Y	Y	Y	Y	Y	Y	Y	Y	Y	Y	Y	Y	Y	Y	Y	Y	Y
fes	Y	Y	Y	Y	Y	Y	Y	Y	Y	Y	Y	Y	Y	Y	Y	Y	Y	Y	Y	Y	Y	Y	Y	Y	Y	Y	Y	Y
fimA	Y	Y	Y	Y	Y	Y	Y	Y	Y	Y	Y	Y	Y	Y	Y	Y	Y	Y	Y	Y	Y	Y	Y	Y	Y	Y	Y	Y
fimC	Y	Y	Y	Y	Y	Y	Y	Y	Y	Y	Y	Y	Y	Y	Y	Y	Y	Y	Y	Y	Y	Y	Y	Y	Y	Y	Y	Y
fimD	Y	Y	Y	Y	Y	Y	Y	Y	Y	Y	Y	Y	Y	Y	Y	Y	Y	Y	Y	Y	Y	Y	Y	Y	Y	Y	Y	Y
fimE	Y	Y	Y	Y	Y	Y	Y	Y	Y	Y	Y	Y	Y	Y	Y	Y	Y	Y	Y	Y	Y	Y	Y	Y	Y	Y	Y	Y
fimF	Y	Y	Y	Y	Y	Y	Y	Y	Y	Y	Y	Y	Y	Y	Y	Y	Y	Y	Y	Y	Y	Y	Y	Y	Y	Y	Y	Y
fimG	Y	Y	Y	Y	Y	Y	Y	Y	Y	Y	Y	Y	Y	Y	Y	Y	Y	Y	Y	Y	Y	Y	Y	Y	Y	Y	Y	Y
fimH	Y	Y	Y	Y	Y	Y	Y	Y	Y	Y	Y	Y	Y	Y	Y	Y	Y	Y	Y	Y	Y	Y	Y	Y	Y	Y	Y	Y
fimI	Y	Y	Y	Y	Y	Y	Y	Y	Y	Y	Y	Y	Y	Y	Y	Y	Y	Y	Y	Y	Y	Y	Y	Y	Y	Y	Y	Y
fyuA	Y	Y	Y	Y	Y	Y	Y	Y	Y	Y	Y	Y	Y	Y	Y	Y	Y	Y	Y	Y	Y	Y	Y	Y	Y	Y	Y	Y
gspL	Y	Y	Y	Y	Y	Y	Y	Y	Y	Y	Y	Y	Y	Y	Y	Y	Y	Y	Y	Y	Y	Y	Y	Y	Y	Y	Y	Y
gspM	Y	Y	Y	Y	Y	Y	Y	Y	Y	Y	Y	Y	Y	Y	Y	Y	Y	Y	Y	Y	Y	Y	Y	Y	Y	Y	Y	Y
irp1	Y	Y	Y	Y	Y	Y	Y	Y	Y	Y	Y	Y	Y	Y	Y	Y	Y	Y	Y	Y	Y	Y	Y	Y	Y	Y	Y	Y
irp2	Y	Y	Y	Y	Y	Y	Y	Y	Y	Y	Y	Y	Y	Y	.	Y	Y	Y	Y	Y	Y	Y	Y	Y	Y	Y	Y	Y
iucA	Y	Y	Y	Y	Y	Y	Y	Y	Y	y	Y	Y	y	Y	Y	Y	.	Y	Y	Y	Y	Y	Y	Y	Y	Y	Y	Y
iucB	Y	Y	Y	Y	Y	Y	Y	Y	Y	Y	Y	Y	Y	Y	Y	Y	.	Y	Y	Y	Y	Y	Y	Y	Y	Y	Y	Y
iucC	Y	Y	Y	Y	Y	Y	Y	Y	Y	Y	Y	Y	Y	Y	Y	Y	.	Y	Y	Y	Y	Y	Y	Y	Y	Y	Y	Y
iucD	Y	Y	Y	Y	Y	Y	Y	Y	Y	Y	Y	Y	Y	Y	Y	Y	.	Y	Y	Y	Y	Y	Y	Y	Y	Y	Y	Y
iutA	Y	Y	Y	Y	Y	Y	Y	Y	Y	Y	Y	Y	Y	Y	Y	Y	.	Y	Y	Y	Y	Y	Y	Y	Y	Y	Y	Y
kpsD	Y	Y	Y	Y	Y	Y	Y	Y	Y	Y	Y	Y	Y	Y	Y	Y	Y	Y	Y	Y	Y	Y	Y	Y	Y	Y	Y	Y
kpsM	Y	Y	Y	Y	Y	Y	Y	Y	Y	Y	Y	Y	Y	Y	Y	Y	Y	Y	Y	Y	Y	Y	Y	Y	Y	Y	Y	Y
ompA	Y	Y	Y	Y	Y	Y	Y	Y	Y	Y	Y	Y	Y	Y	Y	Y	Y	Y	Y	Y	Y	Y	Y	Y	Y	Y	Y	Y
papB	Y	Y	Y	Y	Y	Y	Y	Y	Y	Y	Y	Y	Y	Y	Y	Y	Y	Y	Y	Y	Y	Y	Y	Y	Y	Y	Y	Y
papI	Y	Y	Y	Y	Y	Y	Y	Y	Y	Y	Y	Y	Y	Y	Y	Y	Y	Y	Y	Y	Y	Y	Y	Y	Y	Y	Y	Y

Continuation Table S5. Virulence factors/determinants found in common in all isolates.

AB factor	R1	R2	R3	R4	R5	R6	R7	R8	R9	R10	R11	R12	R13	R14	S1	S2	S3	S4	S5	S6	S7	S8	S9	S10	S11	S12	S13	S14
papX	Y	Y	Y	Y	Y	Y	Y	Y	Y	Y	Y	Y	Y	Y	Y	Y	Y	Y	Y	Y	Y	Y	Y	Y	Y	Y	Y	Y
sat	Y	Y	Y	Y	Y	Y	Y	Y	Y	Y	Y	Y	Y	Y	Y	Y	.	Y	Y	Y	Y	Y	Y	Y	Y	Y	Y	Y
yagV/ecpE	Y	Y	Y	Y	Y	Y	Y	Y	Y	Y	Y	Y	Y	Y	Y	Y	Y	Y	Y	Y	Y	Y	Y	Y	Y	Y	Y	Y
yagW/ecpD	Y	Y	Y	Y	Y	Y	Y	Y	Y	Y	Y	Y	Y	Y	Y	Y	Y	Y	Y	Y	Y	Y	Y	Y	Y	Y	Y	Y
yagX/ecpC	Y	Y	Y	Y	Y	Y	Y	Y	Y	Y	Y	Y	Y	Y	Y	Y	Y	Y	Y	Y	Y	Y	Y	Y	Y	Y	Y	Y
yagY/ecpB	Y	Y	Y	Y	Y	Y	Y	Y	Y	Y	Y	Y	Y	Y	Y	Y	Y	Y	Y	Y	Y	Y	Y	Y	Y	Y	Y	Y
yagZ/ecpA	Y	Y	Y	Y	Y	Y	Y	Y	Y	Y	Y	Y	Y	Y	Y	Y	Y	Y	Y	Y	Y	Y	Y	Y	Y	Y	Y	Y
ybtA	Y	Y	Y	Y	Y	Y	Y	Y	Y	Y	Y	Y	Y	Y	Y	Y	Y	Y	Y	Y	Y	Y	Y	Y	Y	Y	Y	Y
ybtE	Y	Y	Y	Y	Y	Y	Y	Y	Y	Y	Y	Y	Y	Y	Y	Y	Y	Y	Y	Y	Y	Y	Y	Y	Y	Y	Y	Y
ybtP	Y	Y	Y	Y	Y	Y	Y	Y	Y	Y	Y	Y	Y	Y	Y	Y	Y	Y	Y	Y	Y	Y	Y	Y	Y	Y	Y	Y
ybtQ	Y	Y	Y	Y	Y	Y	Y	Y	Y	Y	Y	Y	Y	Y	Y	Y	Y	Y	Y	Y	Y	Y	Y	Y	Y	Y	Y	Y
ybtS	Y	Y	Y	Y	Y	Y	Y	Y	Y	Y	Y	Y	Y	Y	Y	Y	Y	Y	Y	.	Y	Y	Y	Y	Y	Y	Y	Y
ybtT	Y	Y	Y	Y	Y	Y	Y	Y	Y	Y	Y	Y	Y	Y	Y	Y	Y	Y	Y	Y	Y	Y	Y	Y	Y	Y	Y	Y
ybtU	Y	Y	Y	Y	Y	Y	Y	Y	Y	Y	Y	Y	Y	Y	Y	Y	Y	Y	Y	Y	Y	Y	Y	Y	Y	Y	Y	Y
ybtX	Y	Y	Y	Y	Y	Y	Y	Y	Y	Y	Y	Y	Y	Y	Y	Y	Y	Y	Y	Y	Y	Y	Y	Y	Y	Y	Y	Y
ykgK/ecpR	Y	Y	Y	Y	Y	Y	Y	Y	Y	Y	Y	Y	Y	Y	Y	Y	Y	Y	Y	Y	Y	Y	Y	Y	Y	Y	Y	Y

Table S6. Antimicrobial resistance factors found in common in all isolates.

AB factor	R1	R2	R3	R4	R5	R6	R7	R8	R9	R10	R11	R12	R13	R14	S1	S2	S3	S4	S5	S6	S7	S8	S9	S10	S11	S12	S13	S14
**CRP**	Y	Y	Y	Y	Y	Y	Y	Y	Y	Y	Y	Y	Y	Y	Y	Y	Y	Y	Y	Y	Y	Y	Y	Y	Y	Y	Y	Y
**CTX-M-15**	Y	Y	Y	Y	Y	Y	Y	Y	Y	Y	Y	Y	Y	Y	Y	Y	Y	Y	Y	Y	Y	Y	Y	Y	Y	Y	Y	Y
***E. coli* acrA**	Y	Y	Y	Y	Y	Y	Y	Y	Y	Y	Y	Y	Y	Y	Y	Y	Y	Y	Y	Y	Y	Y	Y	Y	Y	Y	Y	Y
***E. coli* ampC**	Y	Y	Y	Y	Y	Y	Y	Y	Y	Y	Y	Y	Y	Y	Y	Y	Y	Y	Y	Y	Y	Y	Y	Y	Y	Y	Y	Y
***E. coli* ampH**	Y	Y	Y	Y	Y	Y	Y	Y	Y	Y	Y	Y	Y	Y	Y	Y	Y	Y	Y	Y	Y	Y	Y	Y	Y	Y	Y	Y
***E. coli* emrE**	Y	Y	Y	Y	Y	Y	Y	Y	Y	Y	Y	Y	Y	Y	Y	Y	Y	Y	Y	Y	Y	Y	Y	Y	Y	Y	Y	Y
***E. coli* mdfA**	Y	Y	Y	Y	Y	Y	Y	Y	Y	Y	Y	Y	Y	Y	Y	Y	Y	Y	Y	Y	Y	Y	Y	Y	Y	Y	Y	Y
**H-NS**	Y	Y	Y	Y	Y	Y	Y	Y	Y	Y	Y	Y	Y	Y	Y	Y	Y	Y	Y	Y	Y	Y	Y	Y	Y	Y	Y	Y
**acrB**	Y	Y	Y	Y	Y	Y	Y	Y	Y	Y	Y	Y	Y	Y	Y	Y	Y	Y	Y	Y	Y	Y	Y	Y	Y	Y	Y	Y
**acrD**	Y	Y	Y	Y	Y	Y	Y	Y	Y	Y	Y	Y	Y	Y	Y	Y	Y	Y	Y	Y	Y	Y	Y	Y	Y	Y	Y	Y
**acrE**	Y	Y	Y	Y	Y	Y	Y	Y	Y	Y	Y	Y	Y	Y	Y	Y	Y	Y	Y	Y	Y	Y	Y	Y	Y	Y	Y	Y
**acrF**	Y	Y	Y	Y	Y	Y	Y	Y	Y	Y	Y	Y	Y	Y	Y	Y	Y	Y	Y	Y	Y	Y	Y	Y	Y	Y	Y	Y
**acrS**	Y	Y	Y	Y	Y	Y	Y	Y	Y	Y	Y	Y	Y	Y	Y	Y	Y	Y	Y	Y	Y	Y	Y	Y	Y	Y	Y	Y
**bacA**	Y	Y	Y	Y	Y	Y	Y	Y	Y	Y	Y	Y	Y	Y	Y	Y	Y	Y	Y	Y	Y	Y	Y	Y	Y	Y	Y	Y
**baeR**	Y	Y	Y	Y	Y	Y	Y	Y	Y	Y	Y	Y	Y	Y	Y	Y	Y	Y	Y	Y	Y	Y	Y	Y	Y	Y	Y	Y
**baeS**	Y	Y	Y	Y	Y	Y	Y	Y	Y	Y	Y	Y	Y	Y	Y	Y	Y	Y	Y	Y	Y	Y	Y	Y	Y	Y	Y	Y
**cpxA**	Y	Y	Y	Y	Y	Y	Y	Y	Y	Y	Y	Y	Y	Y	Y	Y	Y	Y	Y	Y	Y	Y	Y	Y	Y	Y	Y	Y
**emrA**	Y	Y	Y	Y	Y	Y	Y	Y	Y	Y	Y	Y	Y	Y	Y	Y	Y	Y	Y	Y	Y	Y	Y	Y	Y	Y	Y	Y
**emrB**	Y	Y	Y	Y	Y	Y	Y	Y	Y	Y	Y	Y	Y	Y	Y	Y	Y	Y	Y	Y	Y	Y	Y	Y	Y	Y	Y	Y
**emrK**	Y	Y	Y	Y	Y	Y	Y	Y	Y	Y	Y	Y	Y	Y	Y	Y	Y	Y	Y	Y	Y	Y	Y	Y	Y	Y	Y	Y
**emrR**	Y	Y	Y	Y	Y	Y	Y	Y	Y	Y	Y	Y	Y	Y	Y	Y	Y	Y	Y	Y	Y	Y	Y	Y	Y	Y	Y	Y
**emrY**	Y	Y	Y	Y	Y	Y	Y	Y	Y	Y	Y	Y	Y	Y	Y	Y	Y	Y	Y	Y	Y	Y	Y	Y	Y	Y	Y	Y
**eptA**	Y	Y	Y	Y	Y	Y	Y	Y	Y	Y	Y	Y	Y	Y	Y	Y	Y	Y	Y	Y	Y	Y	Y	Y	Y	Y	Y	Y
**evgA**	Y	Y	Y	Y	Y	Y	Y	Y	Y	Y	Y	Y	Y	Y	Y	Y	Y	Y	Y	Y	Y	Y	Y	Y	Y	Y	Y	Y
**evgS**	Y	Y	Y	Y	Y	Y	Y	Y	Y	Y	Y	Y	Y	Y	Y	Y	Y	Y	Y	Y	Y	Y	Y	Y	Y	Y	Y	Y

Continuation Table S6. Antimicrobial resistance factors found in common in all isolates.

AB factor	R1	R2	R3	R4	R5	R6	R7	R8	R9	R10	R11	R12	R13	R14	S1	S2	S3	S4	S5	S6	S7	S8	S9	S10	S11	S12	S13	S14
**gadW**	Y	Y	Y	Y	Y	Y	Y	Y	Y	Y	Y	Y	Y	Y	Y	Y	Y	Y	Y	Y	Y	Y	Y	Y	Y	Y	Y	Y
**gadX**	Y	Y	Y	Y	Y	Y	Y	Y	Y	Y	Y	Y	Y	Y	Y	Y	Y	Y	Y	Y	Y	Y	Y	Y	Y	Y	Y	Y
**kdpE**	Y	Y	Y	Y	Y	Y	Y	Y	Y	Y	Y	Y	Y	Y	Y	Y	Y	Y	Y	Y	Y	Y	Y	Y	Y	Y	Y	Y
**marA**	Y	Y	Y	Y	Y	Y	Y	Y	Y	Y	Y	Y	Y	Y	Y	Y	Y	Y	Y	Y	Y	Y	Y	Y	Y	Y	Y	Y
**mdtA**	Y	Y	Y	Y	Y	Y	Y	Y	Y	Y	Y	Y	Y	Y	Y	Y	Y	Y	Y	Y	Y	Y	Y	Y	Y	Y	Y	Y
**mdtB**	Y	Y	Y	Y	Y	Y	Y	Y	Y	Y	Y	Y	Y	Y	Y	Y	Y	Y	Y	Y	Y	Y	Y	Y	Y	Y	Y	Y
**mdtC**	Y	Y	Y	Y	Y	Y	Y	Y	Y	Y	Y	Y	Y	Y	Y	Y	Y	Y	Y	Y	Y	Y	Y	Y	Y	Y	Y	Y
**mdtE**	Y	Y	Y	Y	Y	Y	Y	Y	Y	Y	Y	Y	Y	Y	Y	Y	Y	Y	Y	Y	Y	Y	Y	Y	Y	Y	Y	Y
**mdtF**	Y	Y	Y	Y	Y	Y	Y	Y	Y	Y	Y	Y	Y	Y	Y	Y	Y	Y	Y	Y	Y	Y	Y	Y	Y	Y	Y	Y
**mdtG**	Y	Y	Y	Y	Y	Y	Y	Y	Y	Y	Y	Y	Y	Y	Y	Y	Y	Y	Y	Y	Y	Y	Y	Y	Y	Y	Y	Y
**mdtH**	Y	Y	Y	Y	Y	Y	Y	Y	Y	Y	Y	Y	Y	Y	Y	Y	Y	Y	Y	Y	Y	Y	Y	Y	Y	Y	Y	Y
**mdtM**	Y	Y	Y	Y	Y	Y	Y	Y	Y	Y	Y	Y	Y	Y	Y	Y	Y	Y	Y	Y	Y	Y	Y	Y	Y	Y	Y	Y
**mdtN**	Y	Y	Y	Y	Y	Y	Y	Y	Y	Y	Y	Y	Y	Y	Y	Y	Y	Y	Y	Y	Y	Y	Y	Y	Y	Y	Y	Y
**mdtO**	Y	Y	Y	Y	Y	Y	Y	Y	Y	Y	Y	Y	Y	Y	Y	Y	Y	Y	Y	Y	Y	Y	Y	Y	Y	Y	Y	Y
**mdtP**	Y	Y	Y	Y	Y	Y	Y	Y	Y	Y	Y	Y	Y	Y	Y	Y	Y	Y	Y	Y	Y	Y	Y	Y	Y	Y	Y	Y
**msbA**	Y	Y	Y	Y	Y	Y	Y	Y	Y	Y	Y	Y	Y	Y	Y	Y	Y	Y	Y	Y	Y	Y	Y	Y	Y	Y	Y	Y
**pmrF**	Y	Y	Y	Y	Y	Y	Y	Y	Y	Y	Y	Y	Y	Y	Y	Y	Y	Y	Y	Y	Y	Y	Y	Y	Y	Y	Y	Y
**tolC**	Y	Y	Y	Y	Y	Y	Y	Y	Y	Y	Y	Y	Y	Y	Y	Y	Y	Y	Y	Y	Y	Y	Y	Y	Y	Y	Y	Y
**ugd**	Y	Y	Y	Y	Y	Y	Y	Y	Y	Y	Y	Y	Y	Y	Y	Y	Y	Y	Y	Y	Y	Y	Y	Y	Y	Y	Y	Y
**yojI**	Y	Y	Y	Y	Y	Y	Y	Y	Y	Y	Y	Y	Y	Y	Y	Y	Y	Y	Y	Y	Y	Y	Y	Y	Y	Y	Y	Y
**AAC(3)-IId**	.	.	.	.	.	.	.	.	.	.	.	.	.	.	.	.	.	.	.	.	.	.	.	.	.	.	.	Y
**armA**	.	.	.	.	.	.	.	.	.	.	.	.	.	Y	.	.	.	.	.	.	.	.	.	.	.	.	.	.
**ErmB**	.	.	.	.	.	.	.	.	.	.	.	.	.	.	.	.	.	.	.	Y	.	.	.	.	.	.	.	.
**mphE**	.	.	.	.	.	.	.	.	.	.	.	.	.	Y	.	.	.	.	.	.	.	.	.	.	.	.	.	.
**msrE**	.	.	.	.	.	.	.	.	.	.	.	.	.	Y	.	.	.	.	.	.	.	.	.	.	.	.	.	.

Table S7. The number of genes present exclusively in each strain and absent in the rest of isolates.

Isolate	Exclusive genes
R1	119
R2	41
R3	18
R4	8
R5	0
R6	43
R7	1
R8	7
R9	39
R10	5
R11	57
R12	11
R13	2
R14	80
S1	13
S2	0
S3	41
S4	43
S5	12
S6	27
S7	49
S8	4
S9	5
S10	0
S11	0
S12	112
S13	13
S14	67

Figure S1. Distribution of the homologous protein clusters identified in the 28 isolates, based on the number of genomes in which they are present. Core: 28 isoltes; Soft Core: >= 26 isolates; Shell: 3 to 25 isolates; Cloud: <=2 isolates.

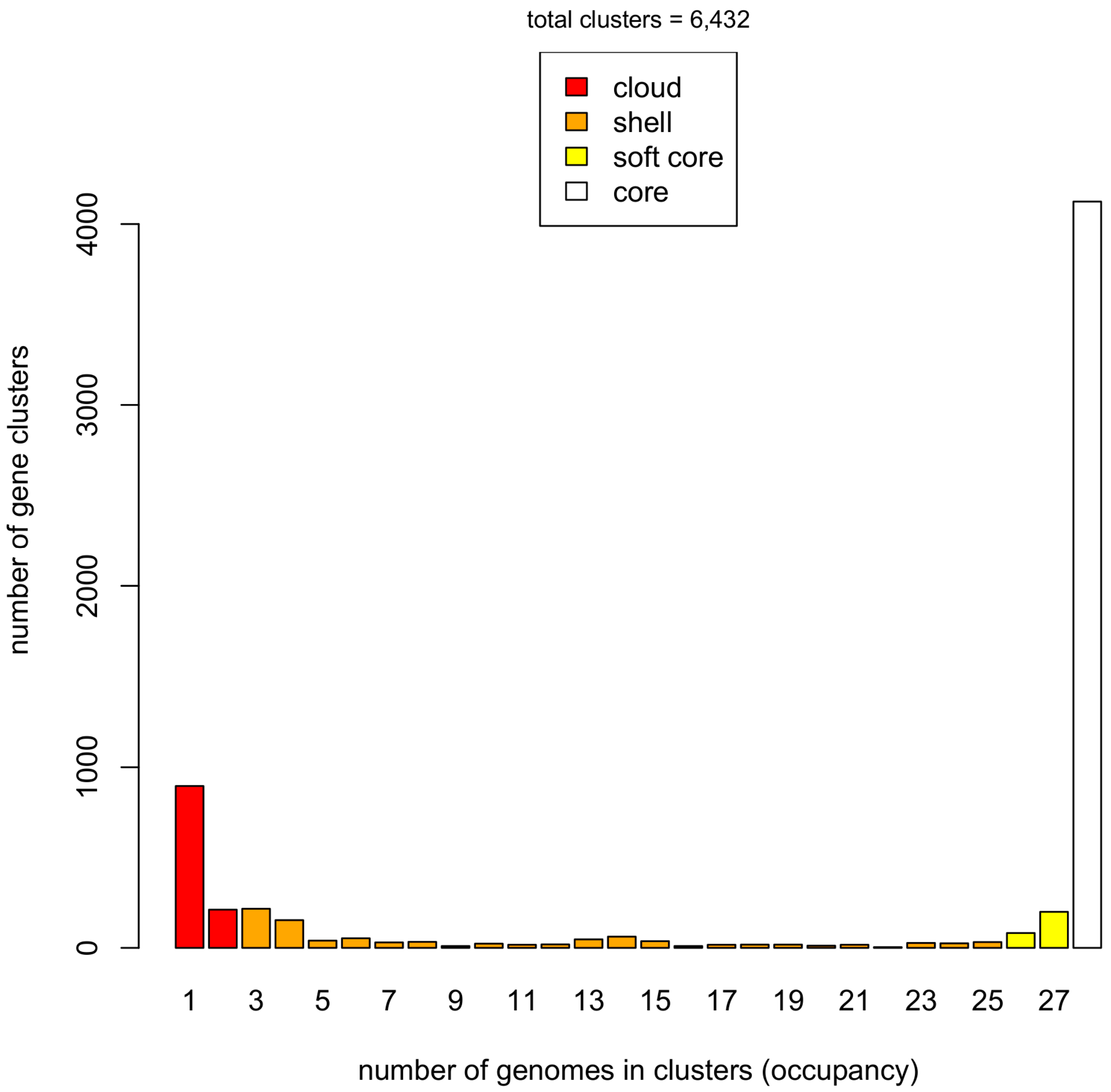

Supplement: Supplementary file 1 [file microorganisms-11-01622-s001.zip › microorganisms-2406198-supplementary material.pdf]
